# Germline variants profiling of *BRCA1* and *BRCA2* in Chinese Hakka breast and ovarian cancer patients

**DOI:** 10.1186/s12885-022-09943-0

**Published:** 2022-08-02

**Authors:** Yunuo Zhang, Heming Wu, Zhikang Yu, Liang Li, Jinhong Zhang, Xinhong Liang, Qingyan Huang

**Affiliations:** 1 Department of Medical Oncology, Meizhou People’s Hospital (Huangtang Hospital), Meizhou Academy of Medical Sciences, Meizhou, China; 2 Center for Precision Medicine, Guangdong Provincial Key Laboratory of Precision Medicine and Clinical Translational Research of Hakka Population, Meizhou People’s Hospital (Huangtang Hospital), Meizhou Academy of Medical Sciences, No 63 Huangtang Road, Meijiang District, Meizhou, 514031 People’s Republic of China; 3 Center for Precision Medicine, Meizhou People’s Hospital (Huangtang Hospital), Meizhou Academy of Medical Sciences, Meizhou, China; 4 Meizhou Municipal Engineering and Technology Research Center for Molecular Diagnostics of Major Genetic Disorders, Meizhou People’s Hospital (Huangtang Hospital), Meizhou Academy of Medical Sciences, Meizhou, China; 5 Radiology department, Meizhou People’s Hospital (Huangtang Hospital), Meizhou Academy of Medical Sciences, Meizhou, China

**Keywords:** *BRCA* gene, Breast cancer, Ovarian cancer, Variants, Hakka population

## Abstract

**Objective:**

To investigate the prevalence and spectrum of *BRCA1* and *BRCA2* mutations in Chinese Hakka patients with breast and ovarian cancer.

**Methods:**

A total of 1,664 breast or ovarian cancer patients were enrolled for genetic testing at our hospital. Germline mutations of the *BRCA* gene were analysed by next-generation sequencing, including the coding regions and exon intron boundary regions.

**Results:**

The 1,664 patients included 1,415 (85.04%) breast cancer patients and 245 (14.72%) ovarian cancer patients, while four (0.24%) patients had both the breast and ovarian cancers. A total of 151 variants, including 71 *BRCA1* variants and 80 *BRCA2* variants, were detected in the 234 (14.06%) patients. The 151 variants included 58 pathogenic variants, 8 likely pathogenic variants, and 85 variants of unknown significance (VUS). A total of 56.25% (18/32) and 65.38% (17/26) of pathogenic variants (likely pathogenic variants are not included) were distributed in exon 14 of *BRCA1* and exon 11 of *BRCA2*, respectively. The most common pathogenic variants among this Hakka population are c.2635G > T (p.Glu879*) (*n* = 7) in the *BRCA1* gene and c.5164_5165del (p.Ser1722Tyrfs*4) (*n* = 7) in the *BRCA2* gene among the Hakka population. A hotspot mutation in the Chinese population, the *BRCA1* c.5470_5477del variant was not found in this Hakka population. The prevalence and spectrum of variants in the *BRCA* genes in the Hakka patients are different from that in other ethnic groups.

**Conclusions:**

The most common pathogenic variant in this population is c.2635G > T in the *BRCA1* gene, and c.5164_5165delAG in the *BRCA2* gene in this population. The prevalence and spectrum of variants in the *BRCA1* and *BRCA2* genes in the Hakka patients from southern China are different from those in other ethnic groups.

**Supplementary Information:**

The online version contains supplementary material available at 10.1186/s12885-022-09943-0.

## Introduction

With the development of the economy and society, women are increasingly stressed at work and in their personal lives. Additionally, and the incidence of breast cancer and ovarian cancer is on the increasing [1]. Worldwide, breast cancer has surpassed lung cancer as the most common cancer in women, and it is the leading cause of cancer death in females. Ovarian cancer is another one of the most common cancers in women and one of the leading causes of death in women [2]. China is in the stage of cancer transition. The cancer spectrum is changing from developing countries to developed countries, and the burden of breast and ovarian cancer is gradually increasing [3]. Germline mutations in breast cancer susceptibility gene 1 (*BRCA1*) and/or breast cancer susceptibility gene 2 (*BRCA2*) confer an increased risk of breast and ovarian cancers [4].

*BRCA1* is located on chromosome 17, contains 24 exons and encodes a multidomain protein containing 1,863 amino acids [5]. *BRCA2* is located on chromosome 13, contains 27 exons, and encodes a multidomain protein containing 3,418 amino acids [6]. The primary role of the *BRCA1* and *BRCA2* genes is to maintain the integrity of the genome, and they act as tumour suppressor genes [6]. Germline mutations in the *BRCA1* and *BRCA2* genes predispose persons to breast and ovarian cancer [4]. Mutations in the human *BRCA1* and *BRCA2* genes may be race-specific in a given region and region-specific in a given ethnic group [7, 8].

Hakka is a Han ethnic group with a unique genetic background and originates from the Hakka ancestors of the Han nationality in Central China. They migrated southward for many times and united with the ancient Yue residents in Guangdong, Fujian and Jiangxi [9]. Meizhou City is located in the northeastern of Guangdong Province and has a large Hakka population. However, limited information about the *BRCA1* and *BRCA2* mutations in this population is available in databases. This study retrospectively analysed the results of screening for genetic mutations of the *BRCA1* and *BRCA2* in breast and ovarian cancer patients among this population.

## Materials and methods

### Participants

A total of 1,664 breast and/or ovarian cancer patients treated at Meizhou People’s Hospital between May 2017 and June 2021 were enrolled. Inclusion criteria: (1) male or female patients diagnosed with breast cancer; (2) female patients diagnosed with ovarian cancer; and (3) Hakka people based on questionnaires about ethnicity. There were no exclusion criteria. These patients underwent *BRCA1* and *BRCA2* gene germline mutation screening tests. This study was approved by the Ethics Committee of Medicine, Meizhou People's Hospital (Huangtang Hospital), Meizhou Academy of Medical Sciences. All participants signed informed consent in accordance with the Declaration of Helsinki.

### *BRCA1* and *BRCA2* gene mutation screening test using next-generation sequencing (NGS)

A peripheral blood sample (2 mL) was collected from each participant and collected in a tube containing EDTA as an anticoagulant. Genomic DNA was extracted by using the QIAamp DNA Blood Mini Kit (Qiagen, Germany) according to the manufacturer’s instructions. DNA concentration and purity were quantified using a Nanodrop 2000™ Spectrophotometer (ThermoFisher Scientific, Waltham, MA). The DNA samples were sequenced after library construction, template preparation and template enrichment according to the standard operating procedures of the Life Technology Company. Next-generation sequencing was performed on the Ion Proton instrument (Life Technologies) and tested by the CapitalBio Corporation (Beijing, China). The data were analysed by the Torrent Suite 4.4.3 and 5.0.4 (Life Technologies). According to the Human Genome Variation Society (HGVS) guidelines, the genetic variations in this study, were named using the following reference sequences: NM_007294.4 (BRCA1) and NM_000059.4 (BRCA2). There are four grades of variants: pathogenic variants, likely pathogenic variants, variants of uncertain significance (VUS), and likely benign variants.

### Genetic counselling and medical advice

#### Genetic counselling

Counselling before genetic testing needs to clarify the purpose of patient counselling and explain the risks, benefits and limitations of genetic testing to patients. A comprehensive collection of patient family history data was obtained; genetic risk was assessed based on patient specific information. Consultation after genetic testing included interpretation of test results, follow-up preventive measures or treatment strategies, evaluation of patients’ needs and psychological state after learning the results, and timely giving corresponding psychological intervention measures.

### Medical advice

Those patients with negative genetic test results were treated as nonmutant patients and regularly followed up. If the *BRCA1/2* genetics test result was VUS, it was recommended to conduct a *BRCA1/2* genetic test on the immediate relatives of these patients to comprehensively evaluate the possibility of VUS. For patients with pathogenic mutations, it was necessary to explain the risk of carrying mutated genes from other family members and passing them on to future generations. It was recommended to conduct *BRCA1/2* genetic testing for the immediate relatives of these patients.

### Guidance for patient treatment

#### Surgical treatment of cancer patients with BRCA1/2 mutations

Total mastectomy and contralateral prophylactic mastectomy are recommended for *BRCA1/2* mutation patients. However, breast-sparing surgery can be an option for breast cancer patients with *BRCA1/2* mutations. If the lesions of patients with *BRCA1/2* mutant breast cancer are suitable for breast-conserving surgery and the patients are willing to undergo breast-conserving surgery, breast-conserving surgery can be carefully selected on the premise that the risk of ipsilateral breast cancer recurrence/new primary cancer and contralateral breast cancer are informed.

Risk-reducing salpingo-oophorectomy (RRSO) was performed according to the patient's age and *BRCA1/2* gene mutation in ovarian cancer patients. Before RRSO was administered, patients were informed of the common sequelae of iatrogenic menopause, including vasomotor symptoms, osteoporosis, decreased libido, vaginal atrophy and dryness, and cardiovascular disease, as well as the benefits and risks of appropriate remedies.

During routine diagnosis and treatment, we will inform patients of possible surgical options and their risks according to the results of *BRCA1/2* gene mutations. The choice of surgical procedure is up to the patient.

#### Chemotherapy and targeted therapy in cancer patients with BRCA1/2 mutations

Poly ADP-ribose polymerase inhibitors (PARPi) therapy can be used for the treatment of early breast cancer patients with *BRCA1/2* pathogenic mutations, is a providing effective treatment options for early breast cancer patients. After adjuvant chemotherapy, HER-2 negative breast cancer patients with *BRCA1/2* pathogenic mutations may be advised to receive 1 year of Olaparib-targeted therapy postoperatively. PARPi can be used as first-line maintenance therapy for ovarian cancer patients with *BRCA1/2* mutations. In addition, Carboplatin may be recommended for advanced triple-negative breast cancer patients with *BRCA1/2* mutations.

In clinical treatment, the selection of chemotherapy drugs or targeted drugs needs to be considered comprehensively according to the patient's condition. In the case of informed consent, it is up to the patient to decide which treatment option to choose.

### Statistical analyses

SPSS statistical software version 21.0 was used for data analyses. Continuous variable data are represented as the mean ± SD. Descriptive analysis was used to show the proportions of sex, different age groups, and disease types in subjects, and to compare the frequencies of the *BRCA*1 and *BRCA2* variants among different populations.

## Results

### Population characteristics

A total of 1,664 breast or ovarian cancer patients were included in the present study, including 1,661 (99.8%) women and 3 (0.2%) men. There were 76 patients (4.6%) under the age of 35, 749 cases (45.0%) between the ages of 35 and 50, and 839 cases (50.4%) beyond the age of 50. The mean ages of patients in the < 35, 35–50, and > 50 years age groups were 29.88 ± 4.60, 44.13 ± 4.34 and 58.23 ± 5.96 years, respectively. There were 1,415 patients (85.04%) with breast cancer, 245 patients (14.72%) with ovarian cancer, and 4 patients (0.24%) with both breast and ovarian cancers. The mean ages of patients with breast cancer, ovarian cancer, and both breast and ovarian cancers were 50.03 ± 9.17, 53.78 ± 12.15 and 56.00 ± 8.29 years, respectively. There were 882 (53.0%) patients in clinical stage 0-II, and 717 (43.1%) patients in clinical stage III-IV (Table 1). The results showed that these patients were roughly evenly divided between those under 50 years old and those over 50 years old, and the majority of these patients were breast cancer patients.

**Table 1:** Clinical characteristics of breast cancer and ovarian cancer patients

Characteristics	Number (Mean ± SD)	Percentage (%)
Gender		
Female	1,661	99.8
Male	3	0.2
Age (years)		
< 35	76 (29.88 ± 4.60)	4.6
35–50	749 (44.13 ± 4.34)	45.0
> 50	839 (58.23 ± 5.96)	50.4
Type of cancer		
Breast cancer only	1,415	85.04
Ovarian cancer only	245	14.72
Both breast and ovarian cancer	4	0.24
Mean age of breast cancer (years)	50.03 ± 9.17	
Mean age of ovarian cancer (years)	53.78 ± 12.15	
Mean age of both breast and ovarian cancer (years)	56.00 ± 8.29	
Clinical stage		
0-II	882	53.0
III-IV	717	43.1
Unknown	65	3.9

### Frequency and distribution of *BRCA1* and *BRCA2* variants in the Hakka population

**Fig. 1:**
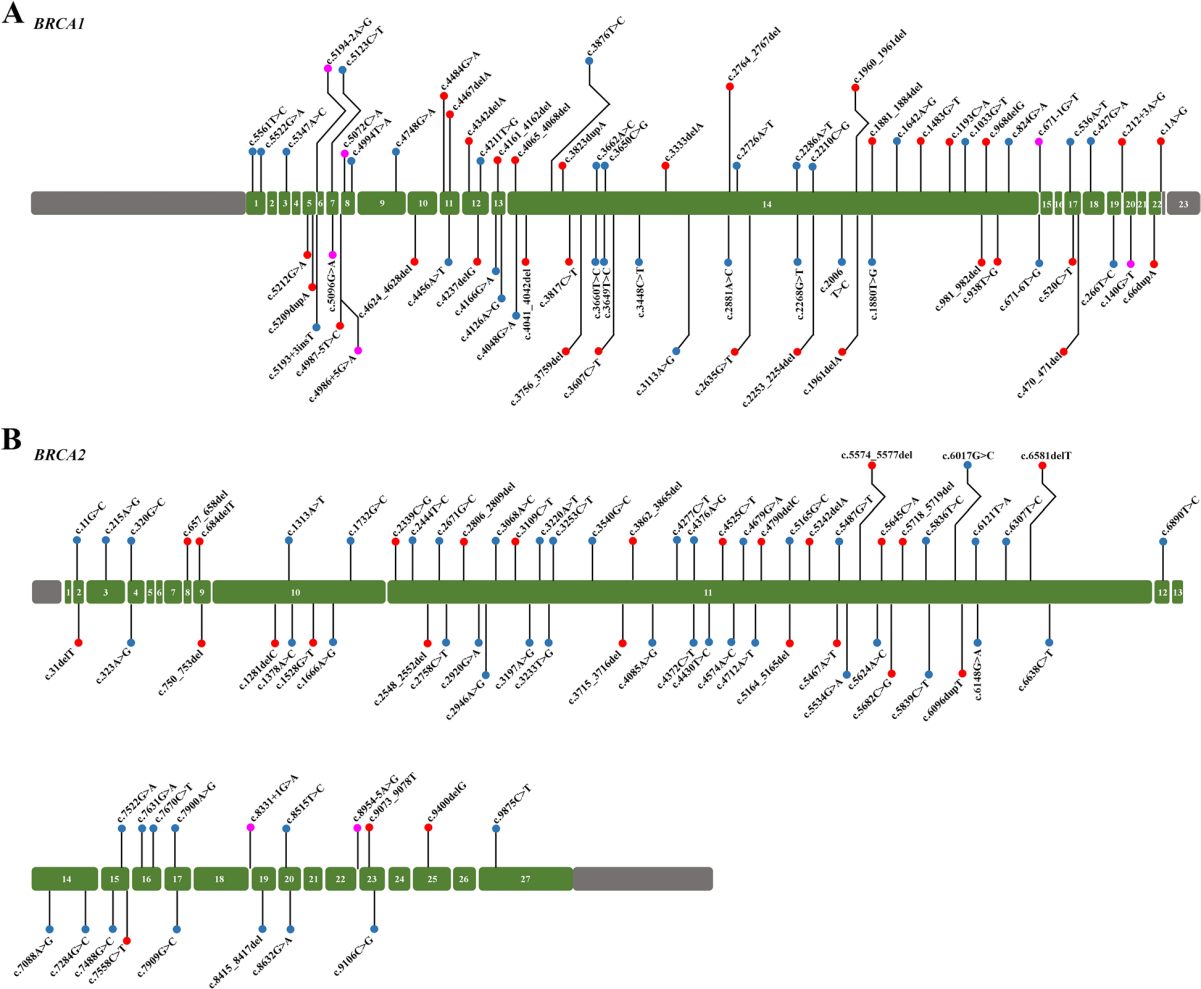
The distribution of various types of variants in the exons of *BRCA1* (**A**) and *BRCA2* (**B**) exons. Grey boxes represent untranslated regions and green boxes represent coding exons. The positions on the gene map indicate the locations of the mutations and the changes in base (red circle: pathogenic variant; pink circle: likely pathogenic variant; blue circle: variant of uncertain significance)

There were 234 patients (234/1,664, 14.06%) with *BRCA* gene variants (including pathogenic variants, likely pathogenic variants, and VUS). Among these patients, 125 patients (125/234, 53.42%) had *BRCA1* gene variant/variants, 101 patients (101/234, 43.16%) had *BRCA2* gene variant/variants, and 8 patients (8/234, 3.42%) had both *BRCA1* and *BRCA2* gene variants. A total of 151 variants of the *BRCA* gene (71 *BRCA1* variants and 80 *BRCA2* variants; including 58 pathogenic variants, 8 likely pathogenic variants, 85 variants of unknown significance (VUS)) were detected. Variants were detected in all exons of the *BRCA1* gene except exons 2, 4, 6, 15, 16 and 21 (Fig. 1A). Variants were detected in all exons of the *BRCA2* gene except exons 1, 5, 6, 7, 13, 18, 21, 22, 24 and 26 (Fig. 1B). There were 102 patients (102/1,664, 6.13%) with pathogenic and likely pathogenic variants of the *BRCA* gene, including 90 patients (90/1,664, 5.41%) with pathogenic variants, and 12 patients (12/1,664, 0.72%) with likely pathogenic variants.

### Recurrent variants in the *BRCA1* and *BRCA2* genes in the Hakka population

**Fig. 2:**
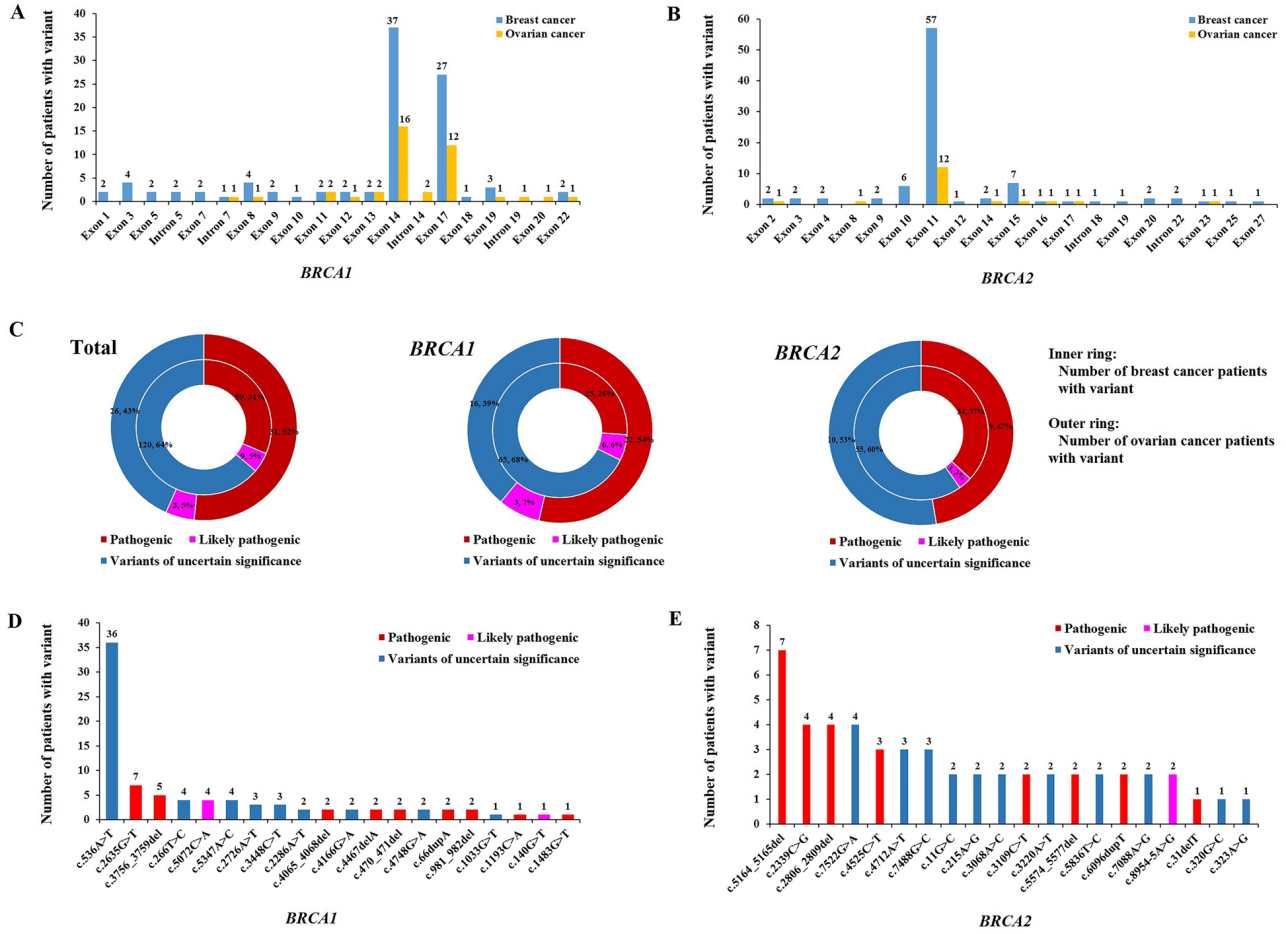
Recurrent variants in *BRCA1* and *BRCA2* genes in Hakka population. Interpretation of *BRCA1* (**A**) and *BRCA2* (**B**) variant carrier number of breast cancer and ovarian cancer patients. The number of different variants for the number of variant carriers of breast cancer patients (inner ring) and the number of variant carriers of ovarian cancer patients (outer ring) (**C**). The top 25 variant types of *BRCA1* (**D**) and *BRCA2* (**E**) in descending order in the Hakka population

While 118 of the 151 distinct *BRCA* variants were observed only once in a patient, 33 *BRCA* variants were detected in multiple patients (at least two or more patients). Variants in *BRCA1* exon 14 were detected in 37 breast cancer patients and 16 ovarian cancer patients; this was the most frequently mutated exon of *BRCA1*. The next most common exon of *BRCA1* with variants was exon 17 (27 breast cancer patients and 12 ovarian cancer patients) (Fig. 2A). Variants in exon 11 of *BRCA2* were detected in 57 breast cancer patients and 12 ovarian cancer patients; this was the most frequently mutated exon of *BRCA2*. The next most common exons of *BRCA2* with variants were exon 15 (7 breast cancer patients and 1 ovarian cancer patient) and exon 10 (6 breast cancer patients) (Fig. 2B). There were 25 breast cancer patients with pathogenic variants, 6 with likely pathogenic variants, and 65 with VUS in the *BRCA1* gene. There were 22 ovarian cancer patients with pathogenic variants, 3 with likely pathogenic variants, and 16 with VUS in the *BRCA1* gene. There were 34 breast cancer patients with pathogenic variants, 3 with likely pathogenic variants, and 55 with VUS in the *BRCA2* gene. There were 9 ovarian cancer patients with pathogenic variants and 10 with VUS in the *BRCA2* gene (Fig. 2C).

The c.536A > T variant (p.Tyr179Phe, VUS) (*n* = 36) and c.2635G > T variant (p.Glu879*, pathogenic) (*n* = 7) in the *BRCA1* gene and the c.5164_5165del variant (p.Ser1722Tyrfs*4, pathogenic) (*n* = 7), c.2339C > G variant (p.Ser780*, pathogenic) (*n* = 4), and c.2806_2809del variant (p.Ala938Profs*21, pathogenic) (*n* = 4) in the *BRCA2* gene were the most common variants in the Hakka population. The most common pathogenic variant in the *BRCA1* gene was c.2635G > T (p.Glu879*) (*n* = 7), and the most common pathogenic variant in the *BRCA2* gene was c.5164_5165del (p.Ser1722Tyrfs*4) (*n* = 7) (Fig. 2D and E). The detailed information for each variant, including mutation site, amino acid change, and number of patients detected for each mutation in the *BRCA* gene, is provided in Table 2 (*BRCA1* pathogenic and likely pathogenic variants), Table 3 (*BRCA2* pathogenic and likely pathogenic variants), Supplemental Table 1 (VUS), and Supplemental Table 2 (likely benign variants), respectively.

**Table 2:** The spectrum of *BRCA1* pathogenic and likely pathogenic variants in breast and ovarian cancer patients

Gene	Exon/Intron	Mutation	Amino acid change	ClinVar	Number of patients
*BRCA1*	Exon 5	c.5212G > A	p.Gly1738Arg	Pathogenic	1
*BRCA1*	Exon 5	c.5209dupA	p.Arg1737Lysfs*93	Pathogenic	1
*BRCA1*	Intron 5	c.5194-2A > G	-	Likely pathogenic	1
*BRCA1*	Exon 7	c.5096G > A	p.Arg1699Gln	Likely pathogenic	1
*BRCA1*	Exon 8	c.5072C > A	p.Thr1691Lys	Likely pathogenic	4
*BRCA1*	Intron 7	c.4987-5 T > C	-	Pathogenic	1
*BRCA1*	Intron 7	c.4986 + 5G > A	-	Likely pathogenic	1
*BRCA1*	Exon 10	c.4624_4628del	p.Ser1542Alafs*30	Pathogenic	1
*BRCA1*	Exon 11	c.4484G > A	p.Arg1495Lys	Pathogenic	1
*BRCA1*	Exon 11	c.4467delA	p.Glu1490Asnfs*15	Pathogenic	2
*BRCA1*	Exon 12	c.4342delA	p.Ser1448Alafs*8	Pathogenic	1
*BRCA1*	Exon 12	c.4237delG	p.Glu1413Asnfs*2	Pathogenic	1
*BRCA1*	Exon 13	c.4161_4162del	p.Gln1388Glufs*2	Pathogenic	1
*BRCA1*	Exon 14	c.4065_4068del	p.Asn1355Lysfs*10	Pathogenic	2
*BRCA1*	Exon 14	c.4041_4042del	p.Gly1348Asnfs*7	Pathogenic	1
*BRCA1*	Exon 14	c.3823dupA	p.Ile1275Asnfs*12	Pathogenic	1
*BRCA1*	Exon 14	c.3817C > T	p.Gln1273*	Pathogenic	1
*BRCA1*	Exon 14	c.3756_3759del	p.Ser1253Argfs*10	Pathogenic	5
*BRCA1*	Exon 14	c.3607C > T	p.Arg1203Ter	Pathogenic	1
*BRCA1*	Exon 14	c.3333delA	p.Glu1112Asnfs*5	Pathogenic	1
*BRCA1*	Exon 14	c.2764_2767del	p.Thr922Leufs*77	Pathogenic	1
*BRCA1*	Exon 14	c.2635G > T	p.Glu879*	Pathogenic	7
*BRCA1*	Exon 14	c.2253_2254del	p.Met751Ilefs*10	Pathogenic	1
*BRCA1*	Exon 14	c.1961delA	p.Lys654Serfs*47	Pathogenic	1
*BRCA1*	Exon 14	c.1960_1961del	p.Lys654Valfs*18	Pathogenic	1
*BRCA1*	Exon 14	c.1881_1884del	p.Ser628Glufs*3	Pathogenic	1
*BRCA1*	Exon 14	c.1483G > T	p.Glu495*	Pathogenic	1
*BRCA1*	Exon 14	c.1193C > A	p.Ser398*	Pathogenic	1
*BRCA1*	Exon 14	c.981_982del	p.Cys328*	Pathogenic	2
*BRCA1*	Exon 14	c.968delG	p.Gly323Glufs*18	Pathogenic	1
*BRCA1*	Exon 14	c.938 T > G	p.Leu313*	Pathogenic	1
*BRCA1*	Intron 14	c.671-1G > T	-	Likely pathogenic	1
*BRCA1*	Exon 17	c.520C > T	p.Gln174*	Pathogenic	1
*BRCA1*	Exon 17	c.470_471del	p.Ser157*	Pathogenic	2
*BRCA1*	Intron 19	c.212 + 3A > G	-	Pathogenic	1
*BRCA1*	Exon 20	c.140G > T	p.Cys47Phe	Likely pathogenic	1
*BRCA1*	Exon 22	c.66dupA	p.Glu23Argfs*18	Pathogenic	2
*BRCA1*	Exon 22	c.1A > G	p.Met1Val	Pathogenic	1

**Table 3:** The spectrum of *BRCA2* pathogenic and likely pathogenic variants in breast and ovarian cancer patients

Gene	Exon/Intron	Mutation	Amino acid change	ClinVar	Number of patients
*BRCA2*	Exon 2	c.31delT	p.Phe12Leufs*13	Pathogenic	1
*BRCA2*	Exon 8	c.657_658del	p.Val220Ilefs*4	Pathogenic	1
*BRCA2*	Exon 9	c.684delT	p.Asn228Lysfs*2	Pathogenic	1
*BRCA2*	Exon 9	c.750_753del	p.Asp252Valfs*24	Pathogenic	1
*BRCA2*	Exon 10	c.1281delC	p.Leu428Tyrfs*2	Pathogenic	1
*BRCA2*	Exon 10	c.1528G > T	p.Glu510*	Pathogenic	1
*BRCA2*	Exon 11	c.2339C > G	p.Ser780*	Pathogenic	4
*BRCA2*	Exon 11	c.2548_2552del	p.Phe851Profs*28	Pathogenic	1
*BRCA2*	Exon 11	c.2806_2809del	p.Ala938Profs*21	Pathogenic	4
*BRCA2*	Exon 11	c.3109C > T	p.Gln1037*	Pathogenic	2
*BRCA2*	Exon 11	c.3715_3716del	p.Lys1239Thrfs*3	Pathogenic	1
*BRCA2*	Exon 11	c.3862_3865del	p.Lys1289Alafs*3	Pathogenic	1
*BRCA2*	Exon 11	c.4525C > T	p.Gln1509*	Pathogenic	3
*BRCA2*	Exon 11	c.4790delC	p.Ser1597Phefs*20	Pathogenic	1
*BRCA2*	Exon 11	c.5164_5165del	p.Ser1722Tyrfs*4	Pathogenic	7
*BRCA2*	Exon 11	c.5242delA	p.Ser1748Alafs*29	Pathogenic	1
*BRCA2*	Exon 11	c.5467A > T	p.Lys1823*	Pathogenic	1
*BRCA2*	Exon 11	c.5574_5577del	p.Ile1859Lysfs*3	Pathogenic	2
*BRCA2*	Exon 11	c.5645C > A	p.Ser1882*	Pathogenic	1
*BRCA2*	Exon 11	c.5682C > G	p.Tyr1894*	Pathogenic	1
*BRCA2*	Exon 11	c.5718_5719del	p.Leu1908Argfs*2	Pathogenic	1
*BRCA2*	Exon 11	c.6096dupT	p.Ile2033Tyrfs*16	Pathogenic	2
*BRCA2*	Exon 11	c.6581delT	p.Ile2194Metfs*12	Pathogenic	1
*BRCA2*	Exon 15	c.7558C > T	p.Arg2520*	Pathogenic	1
*BRCA2*	Intron 18	c.8331 + 1G > A	-	Likely pathogenic	1
*BRCA2*	Intron 22	c.8954-5A > G	-	Likely pathogenic	2
*BRCA2*	Exon 23	c.9073_9078T	p.Ile3025Phefs*17	Pathogenic	1
*BRCA2*	Exon 25	c.9400delG	p.Gly3134Alafs*29	Pathogenic	1

### Genetic distribution of pathogenic *BRCA1* and *BRCA2* variants

**Fig. 3:**
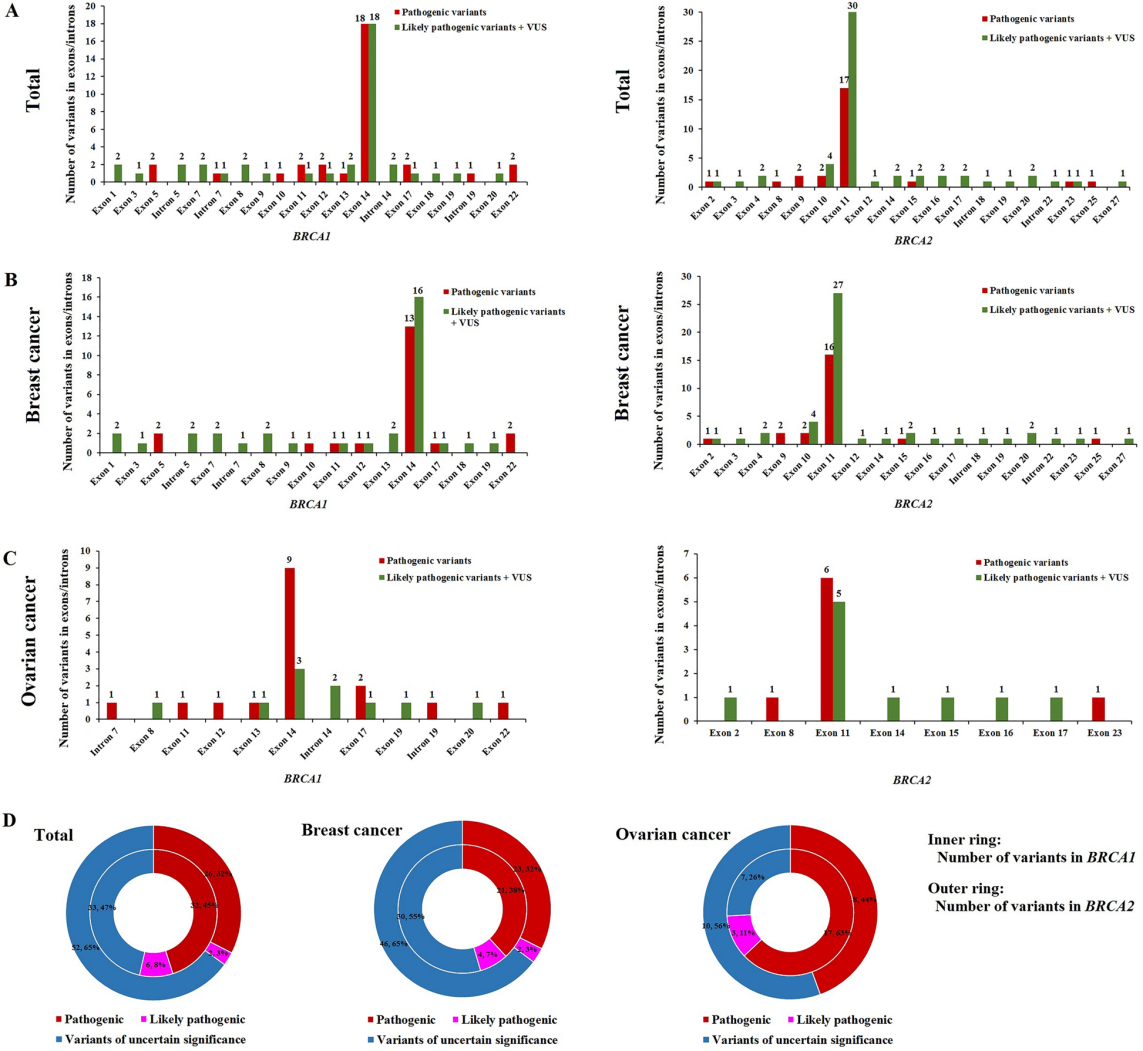
Distribution of pathogenic and nonpathogenic variants in the *BRCA1* and *BRCA2* genes. The number of pathogenic and nonpathogenic variants (likely pathogenic variants + VUS) in each exon and intron of the *BRCA1* and *BRCA2* genes, respectively, among all patients (**A**), breast cancer patients (**B**), and ovarian cancer patients (**C**). The number of different variants in *BRCA1* (inner ring) and *BRCA2* (outer ring) (**D**)

A total of 58 pathogenic variants (32 variants in *BRCA1* gene and 26 variants in *BRCA2*) and 8 likely pathogenic variants were detected in this study. Furthermore, 56.25% (18/32) and 65.38% (17/26) of pathogenic variants were distributed in exon 14 of *BRCA1* and exon 11 of *BRCA2*, respectively (Fig. 3A). In breast cancer patients, there were 61.90% (13/21) and 69.57% (16/23) of pathogenic variants were distributed in exon 14 of *BRCA1* and exon 11 of *BRCA2*, respectively (Fig. 3B). In ovarian cancer patients, there were 52.94% (9/17) and 75.0% (6/8) of pathogenic variants were distributed in exon 14 of *BRCA1* and exon 11 of *BRCA2*, respectively (Fig. 3C).

Among the 151 variants of the *BRCA* gene, 58 distinct pathogenic variants were detected in 90 patients. Among these, 8 likely pathogenic variants (6 variants in *BRCA1* and 2 variants in *BRCA2*) were identified in 12 patients, 85 VUS (33 variants in *BRCA1* and 53 variants in *BRCA2*) were identified in 146 patients, and 25 likely benign variants (6 variants in *BRCA1* and 19 variants in *BRCA2*) were identified in 237 patients. In breast cancer patients, there were 21 pathogenic variants, 4 likely pathogenic variants, and 30 VUS in *BRCA1*, and 23 pathogenic variants, 2 likely pathogenic variants, and 46 VUS in *BRCA2*. In ovarian cancer patients, there were 17 pathogenic variants, 3 likely pathogenic variants, and 7 VUS in *BRCA1* and 8 pathogenic variants and 10 VUS in the *BRCA2* (Fig. 3D).

### Ethnicity comparison of *BRCA1* and *BRCA2* pathogenic variants

The high frequency of *BRCA1* and *BRCA2* variants in the Hakka population was analysed and compared with those from other ethnicities. The most common variants in *BRCA1* and *BRCA2* among the Hakka, Chinese, other Asian, European, Latin American, Caribbean, and African populations are illustrated in Table 4. The *BRCA1* c.68_69delAG was the most pathogenic variant in the Indian population [10], Ashkenazi Jewish population [11], Ashkenazi Jewish population in Argentina [12], Peruvian population [13], South African Indian population [14] and South African population [15]. *BRCA1* c.5266dupC was the most pathogenic variant in the Polish population [16], Italian population [17], and Southern Brazilian population [18]. There were different hotspot mutations among other populations. They are as follows: *BRCA1* c.5251C > T and c.4997dup in the Vietnamese population [19]; *BRCA1* c.4508C > A, c.4065_4068delTCAA, and *BRCA2* c.3109C > T, c.4829_4830delTG in the Pakistani population [20]; *BRCA1* c.390C > A, c.3627dupA, and *BRCA2* c.7480C > T, c.1399A > T in the Korean population [21]; *BRCA1* c.5123C > A, c.211A > G, and *BRCA2* c.2806_2809delAAAC, c.6024dupG in the Spanish population [22]; *BRCA1* c.5123C > A, and *BRCA2* c.6174delT in the Latin American and the Caribbean populations [23]; and *BRCA1* c.211dupA, c.798_799delTT, and *BRCA2* c.1310_1313delAAGA in the North African population [24]. In a recent meta-analysis of *BRCA1* and *BRCA2* gene variations in Chinese individuals, c.5470_5477delATTGGGCA, c.2612C > T, and c.3548A > G in *BRCA1*, and c.3109C > T, c.2806_2809delAAAC, and c.5164_5165delAG in *BRCA2* were the most common variants [25]. The most common pathogenic variants were c.2635G > T, c.3756_3759delGTCT, and c.4065_4068delTCAA in the *BRCA1* gene and c.5164_5165del, c.2339C > G, and c.2806_2809delACAA in the *BRCA2* gene among the Hakka population, respectively. These results showed that the hotspots of pathogenic variants in the *BRCA* genes demonstrate showed race-specific and region-specific differences.

**Table 4:** Comparison of the *BRCA* pathogenic variants in the populations of Hakka population and other populations at home and abroad

Population	*BRCA1*			*BRCA2*			Ref
	First	Second	Third	First	Second	Third	
Asian							
Our data (Hakka)	c.2635G > T	c.3756_3759delGTCT	c.4065_4068delTCAA	c.5164_5165delAG	c.2339C > G	c.2806_2809delACAA	
Chinese	c.5470_5477delATTGGGCA	c.2612C > T	c.3548A > G	c.3109C > T	c.2806_2809delAAAC	c.5164_5165delAG	[25]
Vietnamese	c.5251C > T	c.4997dup		No hotspot			[19]
Indian	c.68_69delAG	c.5074 + 1G > A	c.3607C > T	c.5722_5723delCT			[10]
Pakistani	c.4508C > A	c.4065_4068delTCAA	c.68_69delAG	c.3109C > T	c.4829_4830delTG		[20]
Korean	c.390C > A	c.3627dupA	c.922_924delAGCinsT	c.7480C > T	c.1399A > T	c.5576_5579delTTAA	[21]
European							
Ashkenazi Jewish	c.68_69delAG	c.5266dupC		c.5946delT			[11]
Polish	c.5266dupC	c.181 T > G	c.5251C > T	-			[16]
Spanish	c.5123C > A	c.211A > G	-	c.2806_2809delAAAC	c.6024dupG	c.6275_6276delTT	[22]
Italian	c.5266dupC	c.2406_2409delGAGT	c.5062_5064delGTT	c.6313delA	c.5722_5723delCT	-	[17]
Latin America and the Caribbean populations	c.5123C > A	-	-	c.6174delT	-	-	[23]
Ashkenazi Jewish in Argentina	c.68_69delAG	c.5266dupC	-	c.5946delT	-	-	[12]
Southern Brazilian	c.5266dupC	c.5177_5180delGAAA	c.5251C > T	c.2808_2811delACAA	c.1138delA	-	[18]
Peruvian	c.68_69delAG	-	-	c.2808_2811delACAA	-	-	[13]
African							
South African Indian	c.68_69delAG	c.4308_4309delTT	-	c.8754 + 1G > A	c.4003G > T	-	[14]
South African	c.68_69delAG	c.1374delC	c.2641G > T	c.7934delG	c.5771_5774del	c.6448_6449dup	[15]
North African	c.211dupA	c.798_799delTT	c.5266dupC	c.1310_1313delAAGA	-	-	[24]

## Discussion

The *BRCA* genes are an important genes that determines the genetic susceptibility to cancer by participating in the regulation of DNA damage and repair, cell growth and apoptosis and by playing an indispensable role in maintaining the genetic stability of cells [26, 27]. Variants in the *BRCA* genes can lead to breast and ovarian cancer. Screening for *BRCA* gene mutations can effectively assess and predict the risk for breast and ovarian cancer. Thus, they can indicate the appropriate intervention to reduce the incidence of the disease and guide a precise treatment.

There are relatively few complete data on *BRCA* gene mutations in the Chinese population. At present, there is a gap in research on *BRCA* mutations in breast cancer and ovarian cancer patients in the Chinese population. Both of the *BRCA1* and *BRCA2* gene fragments are relatively long, with many diverse variants dispersed throughout the genes. Mutation types in different populations vary greatly, making it difficult to identify specific hotspot mutations. Studies have found that certain mutations are more common in certain populations, known as the founder effect, and these are called founder mutations. *BRCA* founder mutations have been identified in some ethnic groups worldwide. For example, *BRCA1* c.68_69delAG, *BRCA1* c.5266dupC and *BRCA2* c.5946delT in Ashkenazi Jews [11], and *BRCA1* c.5266dupC and *BRCA1* c.4035delA are common in Polish patients [28]. The most common pathogenic variant in *BRCA1* was c.981_982delAT, and in *BRCA2* c.3195_3198delTAAT [29]. The c.303 T > G, c.1623dupG, and c.4122_4123delTG variants in *BRCA1* are frequently found in the African patients with breast cancer [30]. The c.5266dupC, c.5177_5180delGAAA, and c.5251C > T variants in *BRCA1* and the c.2808_2811delACAA and c.1138delA variants in *BRCA2* were the most common variants among breast and ovarian cancer patients from Brazil [18]. *BRCA1* ex9-12del is the most common variant in Mexican patients [31], and *BRCA1* c.5095C > T is the most common variant in Arab breast and ovarian cancer patients [32]. *BRCA1* c.68_69delAG is the most common variant in South Asian patients [33] and Latina patients residing in southern California [34]. *BRCA2* c.3922G > T is a founder mutation in the Puerto Rican population [35]. The *BRCA1* c.5266dupC mutation is recorded as the founder mutation in Italian [36], Northeastern Romanian [37], and Turkish populations [38]. *BRCA1* c.5266dupC and c.181 T > G are founder mutations in the Polish population [39]. *BRCA1* c.3319G > T is a founder mutation in the Western Denmark [40]. Slavic *BRCA1* and *BRCA2* founder mutations include *BRCA1* c.5266dupC, *BRCA1* c.4034delA, and *BRCA1* c.68_69delAG [41]. *BRCA1* c.4136_4137delCT and c.1140dupG are founder mutations in the Middle Eastern population [42]. *BRCA1* c.798_799delTT is a founder mutation in the North African population [43].

In 2016, *BRCA1/2* germline mutations were screened in 5,931 unselected Chinese women with breast cancer, and this study found that the *BRCA1* c.5470_5477del was the most common variant in this population [44]. In 2017, Lang et al. enrolled 2,991 breast cancer patients and 1,043 healthy individuals in their study. They found that the most common *BRCA1* mutation was c.5470_5477del, and the most common *BRCA2* mutations were c.470_474del and c.3109C > T [45]. Wang et al. also found that *BRCA1* c.5470_5477del was highly prevalent in a population of Chinese women population [46]. Studies have shown that *BRCA1* c.5470_5477del was a founder mutation in Chinese Han ovarian cancer patients [47] and Chinese Han breast cancer patients [48]. A meta-analysis conducted by Kim et al. on population samples from mainland China in 2016 found that *BRCA1* c.981_982delAT and *BRCA2* c.3195_3198delTAAT were highly prevalent in mainland Chinese population [29]. In 2018, Kwong et al. analysed more than 600 samples from breast cancer patients in Hong Kong and more than 80 samples from Chinese patients who were overseas and found that the *BRCA1* c.964delG and *BRCA2* c.3109C > T mutations are common in the local population of Hong Kong [49]. In a recent meta-analysis of *BRCA1* and *BRCA2* gene variations in Chinese individuals, c.5470_5477del, c.2612C > T, and c.3548A > G in *BRCA1*, and c.3109C > T, c.2806_2809delAAAC, and c.5164_5165delAG in *BRCA2* were the most common variants in this population [25]. In general, *BRCA1* c.5470_5477del is considered to be a hotspot and founder mutation in the Chinese population.

The *BRCA1* c.5470_5477del variant is not found in the Hakka population. Among the Hakka population in this population, the most common *BRCA1* pathogenic variant is c.2635G > T (p.Glu879*) in this study. This variant is predicted to encode a truncated nonfunctional protein. *BRCA1* c.2635G > T, a reported mutation among Hong Kong Chinese patients [50, 51], patients with breast cancer from Malaysia [52], and breast and/or ovarian cancer patients from Singapore [53, 54]. However, this variant is relatively rare in these populations and is not a common variant. This variant is not seen in other populations. Another common mutation *BRCA1* c.3756_3759delGTCT has been detected in some populations, such as Thai [55], Polish [56], Belarusian [57], Italian [58], French-Canadian [59], and Czech populations [60]. *BRCA1* c.4065_4068del has been detected in some populations [61–63]. Another study showed that c.4065_4068del is one of the three most common *BRCA1* variants in Chinese ovarian cancer patients [47]. In the *BRCA2* gene, c.5164_5165delAG has been detected in the Chinese Han population [64], Macau population [65], and Taiwanese populations [66]. *BRCA2* c.2339C > G has been detected in Taiwanese [67], and Japanese [68] individuals. *BRCA2* c.2806_2809del has been detected in Mexican individuals [69].

In addition, there were 3 male breast cancer patients, accounting for 0.21% (3/1430) of the breast cancer patients in this study. Male breast cancer is a rare malignancy that accounts for less than 1% of all breast cancers [70] in some populations. It accounts for 0.48% of cases in the South Korean populations [71], 0.6% in the Australian population [72], 0.9% in the American population [73], and 0.55% in the Danish population [74]. Of course, there are some populations with higher rates of breast cancer in men. For example, the male breast rate is 1.1% in Northern India [75], and it is higher in some populations in Africa (6.2% in North Uganda [76], 2.6% in Burkina Faso [77], and 3.2% in 27 African countries [78]). Epidemiological differences between different groups of people may be related to region, race and living environment. Studies have shown that the major risk factors for the development of male breast cancer include advancing age, hormonal imbalance, radiation exposure, and a family history of breast cancer, but the most relevant risk factor is mutations in the *BRCA2* gene [79, 80]. None of the three male breast cancer patients in this study had *BRCA* mutations. Understanding of the biology, clinical manifestations, genetics and treatment of male breast cancer is evolving, but due to the rarity of the disease, it is not well understood at present. More in-depth research is needed.

In general, the prevalence and spectrum of the *BRCA1* and *BRCA2* genes in the Hakka patients with breast cancer and ovarian cancer from southern China are different from those in other ethnic groups. This study provides a basis and serves as a reference for clinical counselling and the prevention and treatment strategies of breast cancer and ovarian cancer based on genetic screening. Identifying hotspot variants is an effective way to improve genetic counselling because molecular testing can target the hotspot variants, thereby enabling faster and cheaper testing. Clinical *BRCA1* and *BRCA2* testing enables the identification of individuals at elevated risk for hereditary breast and ovarian cancer. The results of this study can provide local patients with more information about pretest and post test genetic testing. Such information includes why it is indicated, possible test outcomes, implications of the test results for family, economic wellbeing, psychosocial wellbeing, and cancer surveillance and prevention options. Thus, genetic counselling was provided to patients.

Although this study has identified some hotspot variants in the Hakka population, we cannot rule out the possibility that other hotspot variants may exist in a larger Hakka patient population. This is one of the limitations of this study. In addition, participants were identified as Hakka through questionnaires, and no population genetic information was collected and analysed on these participants in this study. This is another shortcoming of this study. Finally, in clinical treatment, although the mutation of *BRCA* gene was taken into consideration when choosing treatment options, the correlation between the *BRCA* gene mutation and the prognosis of different treatment options was not analysed. This is one of the deficiencies of this study. In the future, *BRCA* gene mutation studies with a larger sample size should be carried out in China, including multiethnic studies, and unified standards should be adopted to establish a more complete *BRCA* gene mutation database that is consistent with the characteristics of the Chinese population. We believe that this study can complement the *BRCA* gene mutation information in the Chinese population.

## Conclusions

In this study, the *BRCA* gene mutations accounted for a certain proportion of the patients with breast cancer and ovarian cancer in the Hakka population of southern China. In this population, the most common pathogenic variant in the *BRCA1* gene was c.2635G > T, and the most common pathogenic variant in the *BRCA2* gene was c.5164_5165delAG in *BRCA2* gene in this population. The prevalence and spectrum of variants in the *BRCA1* and *BRCA2* genes in the Hakka patients from southern China are different from those in other ethnic groups.

## Supplementary Information

**Additional file 1:** **Supplemental Table 1.** The spectrum of BRCA1 and BRCA2 VUS variants in breast and ovarian cancer patients.

**Additional file 2:** **Supplemental Table 2.** The spectrum of BRCA1 and BRCA2 likely benign variants in breast and ovarian cancer patients.

## Data Availability

The variants generated and/or analysed during the current study are available in the clinVar database (https://www.ncbi.nlm.nih.gov/clinvar/), [the ClinVar accessions for this data are SCV002520768 to SCV002520943].
