# Supplementary material for: Germline variants profiling of BRCA1 and BRCA2 in Chinese Hakka breast and ovarian cancer patients
Source: BMC Cancer. 2022 Aug 2;22:842. doi: 10.1186/s12885-022-09943-0 (PMC9347172; doi:10.1186/s12885-022-09943-0)
Supplement: Supplementary file 1 — Additional file 1: Supplemental Table 1. The spectrum of BRCA1 and BRCA2 VUS variants in breast and ovarian cancer patients. [file 12885_2022_9943_MOESM1_ESM.docx]

Supplemental Table 1 The spectrum of *BRCA1* and *BRCA2* VUS variants in breast and ovarian cancer patients.

| **Gene** | **Exon/Intron** | **Mutation** | **Amino acid change** | **ClinVar** | **Number of patients** |
| --- | --- | --- | --- | --- | --- |
| *BRCA1* | Exon 1 | c.5561T>C | p.Leu1854Pro | VUS | 1 |
| *BRCA1* | Exon 1 | c.5522G>A | p.Ser1841Asn | VUS | 1 |
| *BRCA1* | Exon 3 | c.5347A>C | p.Met1783Leu | VUS | 4 |
| *BRCA1* | Intron 5 | c.5193+3insT | - | VUS | 1 |
| *BRCA1* | Exon 7 | c.5123C>T | p.Ala1708Val | VUS | 1 |
| *BRCA1* | Exon 8 | c.4994T>A | p.Val1665Glu | VUS | 1 |
| *BRCA1* | Exon 9 | c.4748G>A | p.Arg1583Lys | VUS | 2 |
| *BRCA1* | Exon 11 | c.4456A>T | p.Ser1486Cys | VUS | 1 |
| *BRCA1* | Exon 12 | c.4211T>G | p.Leu1404Arg | VUS | 1 |
| *BRCA1* | Exon 13 | c.4166G>A | p.Ser1389Asn | VUS | 2 |
| *BRCA1* | Exon 13 | c.4126A>G | p.Thr1376Ala | VUS | 1 |
| *BRCA1* | Exon 14 | c.4048G>A | p.Gly1350Ser | VUS | 1 |
| *BRCA1* | Exon 14 | c.3876T>C | p.Ser1292Ser | VUS | 1 |
| *BRCA1* | Exon 14 | c.3662A>C | p.Glu1221Ala | VUS | 1 |
| *BRCA1* | Exon 14 | c.3660T>C | p.Asp1220Asp | VUS | 1 |
| *BRCA1* | Exon 14 | c.3650C>G | p.Ser1217Cys | VUS | 1 |
| *BRCA1* | Exon 14 | c.3649T>C | p.Ser1217Pro | VUS | 1 |
| *BRCA1* | Exon 14 | c.3448C>T | p.Pro1150Ser | VUS | 3 |
| *BRCA1* | Exon 14 | c.3113A>G | p.Glu1038Ala | VUS | 1 |
| *BRCA1* | Exon 14 | c.2881A>C | p.Asn961His | VUS | 1 |
| *BRCA1* | Exon 14 | c.2726A>T | p.Asn909Ile | VUS | 3 |
| *BRCA1* | Exon 14 | c.2286A>T | p.Arg762Ser | VUS | 2 |
| *BRCA1* | Exon 14 | c.2268G>T | p.Arg756Ser | VUS | 1 |
| *BRCA1* | Exon 14 | c.2210C>G | p.Thr737Arg | VUS | 1 |
| *BRCA1* | Exon 14 | c.2006T>C | p.Met669Thr | VUS | 1 |
| *BRCA1* | Exon 14 | c.1880T>G | p.Val627Gly | VUS | 1 |
| *BRCA1* | Exon 14 | c.1642A>G | p.Ile548Val | VUS | 1 |
| *BRCA1* | Exon 14 | c.1033G>T | p.Asp345Tyr | VUS | 1 |
| *BRCA1* | Exon 14 | c.824G>A | p.Gly275Asp | VUS | 1 |
| *BRCA1* | Intron 14 | c.671-6T>G | - | VUS | 1 |
| *BRCA1* | Exon 17 | c.536A>T | p.Tyr179Phe | VUS | 36 |
| *BRCA1* | Exon 18 | c.427G>A | p.Glu143Lys | VUS | 1 |
| *BRCA1* | Exon 19 | c.266T>C | p.Ile89Thr | VUS | 4 |
| *BRCA2* | Exon 2 | c.11G>C | p.Gly4Ala | VUS | 2 |
| *BRCA2* | Exon 3 | c.215A>G | p.Asn72Ser | VUS | 2 |
| *BRCA2* | Exon 4 | c.320G>C | p.Arg107Thr | VUS | 1 |
| *BRCA2* | Exon 4 | c.323A>G | p.Asn108Ser | VUS | 1 |
| *BRCA2* | Exon 10 | c.1313A>T | p.Asp438Val | VUS | 1 |
| *BRCA2* | Exon 10 | c.1378A>C | p.Asn460His | VUS | 1 |
| *BRCA2* | Exon 10 | c.1666A>G | p.Asn556Asp | VUS | 1 |
| *BRCA2* | Exon 10 | c.1732G>C | p.Gly578Arg | VUS | 1 |
| *BRCA2* | Exon 11 | c.2444T>C | p.Met815Thr | VUS | 1 |
| *BRCA2* | Exon 11 | c.2671G>C | p.Val891Leu | VUS | 1 |
| *BRCA2* | Exon 11 | c.2758C>T | p.Pro920Ser | VUS | 1 |
| *BRCA2* | Exon 11 | c.2920G>A | p.Asp974Asn | VUS | 1 |
| *BRCA2* | Exon 11 | c.2946A>G | p.Ile982Met | VUS | 1 |
| *BRCA2* | Exon 11 | c.3068A>C | p.Asn1023Thr | VUS | 2 |
| *BRCA2* | Exon 11 | c.3197A>G | p.Asn1066Ser | VUS | 1 |
| *BRCA2* | Exon 11 | c.3220A>T | p.Ser1074Cys | VUS | 2 |
| *BRCA2* | Exon 11 | c.3233T>G | p.Val1078Gly | VUS | 1 |
| *BRCA2* | Exon 11 | c.3253C>T | p.His1085Tyr | VUS | 1 |
| *BRCA2* | Exon 11 | c.3540G>C | p.Lys1180Asn | VUS | 1 |
| *BRCA2* | Exon 11 | c.4085A>G | p.His1362Arg | VUS | 1 |
| *BRCA2* | Exon 11 | c.4277C>T | p.Thr1426Ile | VUS | 1 |
| *BRCA2* | Exon 11 | c.4372C>T | p.His1458Tyr | VUS | 1 |
| *BRCA2* | Exon 11 | c.4376A>G | p.Asn1459Ser | VUS | 1 |
| *BRCA2* | Exon 11 | c.4430T>C | p.Ile1477Thr | VUS | 1 |
| *BRCA2* | Exon 11 | c.4574A>C | p.His1525Pro | VUS | 1 |
| *BRCA2* | Exon 11 | c.4679G>A | p.Ser1560Asn | VUS | 1 |
| *BRCA2* | Exon 11 | c.4712A>T | p.Glu1571Val | VUS | 3 |
| *BRCA2* | Exon 11 | c.5165G>C | p.Ser1722Thr | VUS | 1 |
| *BRCA2* | Exon 11 | c.5487G>T | p.Leu1829Phe | VUS | 1 |
| *BRCA2* | Exon 11 | c.5534G>A | p.Arg1845Lys | VUS | 1 |
| *BRCA2* | Exon 11 | c.5624A>C | p.Lys1875Thr | VUS | 1 |
| *BRCA2* | Exon 11 | c.5836T>C | p.Ser1946Pro | VUS | 2 |
| *BRCA2* | Exon 11 | c.5839C>T | p.Pro1947Ser | VUS | 1 |
| *BRCA2* | Exon 11 | c.6017G>C | p.Ser2006Thr | VUS | 1 |
| *BRCA2* | Exon 11 | c.6121T>A | p.Ser2041Thr | VUS | 1 |
| *BRCA2* | Exon 11 | c.6148G>A | p.Val2050Ile | VUS | 1 |
| *BRCA2* | Exon 11 | c.6307T>C | p.Ser2103Pro | VUS | 1 |
| *BRCA2* | Exon 11 | c.6638C>T | p.Ser2213Phe | VUS | 1 |
| *BRCA2* | Exon 12 | c.6890T>C | p.Ile2297Thr | VUS | 1 |
| *BRCA2* | Exon 14 | c.7088A>G | p.Tyr2363Cys | VUS | 2 |
| *BRCA2* | Exon 14 | c.7284G>C | p.Leu2428Phe | VUS | 1 |
| *BRCA2* | Exon 15 | c.7488G>C | p.Lys2496Asn | VUS | 3 |
| *BRCA2* | Exon 15 | c.7522G>A | p.Gly2508Ser | VUS | 4 |
| *BRCA2* | Exon 16 | c.7631G>A | p.Gly2544Asp | VUS | 1 |
| *BRCA2* | Exon 16 | c.7670C>T | p.Ala2557Val | VUS | 1 |
| *BRCA2* | Exon 17 | c.7900A>G | p.Met2634Val | VUS | 1 |
| *BRCA2* | Exon 17 | c.7909G>C | p.Ala2637Pro | VUS | 1 |
| *BRCA2* | Exon 19 | c.8415_8417del | p.Ser2807del | VUS | 1 |
| *BRCA2* | Exon 20 | c.8515T>C | p.Tyr2839His | VUS | 1 |
| *BRCA2* | Exon 20 | c.8632G>A | p.Glu2878Lys | VUS | 1 |
| *BRCA2* | Exon 23 | c.9106C>G | p.Gln3036Glu | VUS | 1 |
| *BRCA2* | Exon 27 | c.9875C>T | p.Pro3292Leu | VUS | 1 |

VUS, Variant of uncertain significance.
