# Supplementary material for: Germline variants profiling of BRCA1 and BRCA2 in Chinese Hakka breast and ovarian cancer patients
Source: BMC Cancer. 2022 Aug 2;22:842. doi: 10.1186/s12885-022-09943-0 (PMC9347172; doi:10.1186/s12885-022-09943-0)
Supplement: Supplementary file 2 — Additional file 2: Supplemental Table 2. The spectrum of BRCA1 and BRCA2 likely benign variants in breast and ovarian cancer patients. [file 12885_2022_9943_MOESM2_ESM.docx]

Supplemental Table 2 The spectrum of *BRCA1* and *BRCA2* likely benign variants in breast and ovarian cancer patients.

| **Gene** | **Exon/Intron** | **Mutation** | **Amino acid change** | **ClinVar** | **Number of patients** |
| --- | --- | --- | --- | --- | --- |
| *BRCA1* | Exon 14 | c.3739G>A | p.Val1247Ile | Likely benign | 3 |
| *BRCA1* | Exon 14 | c.3596C>T | p.Ala1199Val | Likely benign | 5 |
| *BRCA1* | Exon 14 | c.2566T>C | p.Tyr856His | Likely benign | 40 |
| *BRCA1* | Exon 14 | c.1036C>T | p.Pro346Ser | Likely benign | 4 |
| *BRCA1* | Exon 14 | c.811G>A | p.Val271Met | Likely benign | 6 |
| *BRCA1* | Exon 16 | c.571G>A | p.Val191Ile | Likely benign | 11 |
| *BRCA2* | Exon 5 | c.440A>G | p.Gln147Arg | Likely benign | 5 |
| *BRCA2* | Exon 10 | c.943T>A | p.Cys315Ser | Likely benign | 15 |
| *BRCA2* | Exon 10 | c.1166C>T | p.Pro389Leu | Likely benign | 2 |
| *BRCA2* | Exon 10 | c.1568A>G | p.His523Arg | Likely benign | 14 |
| *BRCA2* | Exon 10 | c.1744A>C | p.Thr582Pro | Likely benign | 4 |
| *BRCA2* | Exon 11 | c.2350A>G | p.Met784Val | Likely benign | 2 |
| *BRCA2* | Exon 11 | c.3445A>G | p.Met1149Val | Likely benign | 1 |
| *BRCA2* | Exon 11 | c.5785A>G | p.Ile1929Val | Likely benign | 17 |
| *BRCA2* | Exon 11 | c.6322C>T | p.Arg2108Cys | Likely benign | 13 |
| *BRCA2* | Exon 11 | c.6325G>A | p.Val2109Ile | Likely benign | 2 |
| *BRCA2* | Exon 13 | c.6941C>T | p.Thr2314Ile | Likely benign | 1 |
| *BRCA2* | Exon 14 | c.7052C>G | p.Ala2351Gly | Likely benign | 6 |
| *BRCA2* | Exon 14 | c.7102T>G | p.Leu2368Val | Likely benign | 6 |
| *BRCA2* | Exon 15 | c.7469T>C | p.Ile2490Thr | Likely benign | 2 |
| *BRCA2* | Exon 18 | c.8090G>A | p.Ser2697Asn | Likely benign | 1 |
| *BRCA2* | Exon 18 | c.8187G>T | p.Lys2729Asn | Likely benign | 26 |
| *BRCA2* | Exon 21 | c.8702G>A | p.Gly2901Asp | Likely benign | 6 |
| *BRCA2* | Exon 27 | c.10150C>T | p.Arg3384* | Likely benign | 1 |
| *BRCA2* | Exon 27 | c.10234A>G | p.Ile3412Val | Likely benign | 44 |
